# Abscisic Acid: A Novel Nutraceutical for Glycemic Control

**DOI:** 10.3389/fnut.2017.00024

**Published:** 2017-06-13

**Authors:** Elena Zocchi, Raquel Hontecillas, Andrew Leber, Alexandra Einerhand, Adria Carbo, Santina Bruzzone, Nuria Tubau-Juni, Noah Philipson, Victoria Zoccoli-Rodriguez, Laura Sturla, Josep Bassaganya-Riera

**Affiliations:** ^1^Department of Experimental Medicine, Section of Biochemistry and Center of Excellence for Biomedical Research, University of Genoa, Genoa, Italy; ^2^BioTherapeutics Inc., Blacksburg, VA, United States; ^3^Nutritional Immunology and Molecular Medicine Laboratory, Biocomplexity Institute of Virginia Tech, Blacksburg, VA, United States

**Keywords:** lanthionine synthetase C-like 2, abscisic acid, diabetes, prediabetes, metabolic syndrome

## Abstract

Abscisic acid is naturally present in fruits and vegetables, and it plays an important role in managing glucose homeostasis in humans. According to the latest U.S. dietary survey, about 92% of the population might have a deficient intake of ABA due to their deficient intake of fruits and vegetables. This review summarizes the *in vitro*, preclinical, mechanistic, and human translational findings obtained over the past 15 years in the study of the role of ABA in glycemic control. In 2007, dietary ABA was first reported to ameliorate glucose tolerance and obesity-related inflammation in mice. The most recent findings regarding the topic of ABA and its proposed receptor lanthionine synthetase C-like 2 in glycemic control and their interplay with insulin and glucagon-like peptide-1 suggest a major role for ABA in the physiological response to a glucose load in humans. Moreover, emerging evidence suggests that the ABA response might be dysfunctional in diabetic subjects. Follow on intervention studies in healthy individuals show that low-dose dietary ABA administration exerts a beneficial effect on the glycemia and insulinemia profiles after oral glucose load. These recent findings showing benefits in humans, together with extensive efficacy data in mouse models of diabetes and inflammatory disease, suggest the need for reference ABA values and its possible exploitation of the glycemia-lowering effects of ABA for preventative purposes. Larger clinical studies on healthy, prediabetic, and diabetic subjects are needed to determine whether addressing the widespread dietary ABA deficiency improves glucose control in humans.

## Introduction

### ABA Is a Conserved Signaling Molecule among Species

ABA is a naturally occurring isoprenoid compound that was originally identified in plants in the 1960s (1). ABA is also present in metazoans, from sponges up to mammals including humans, and so it is well conserved. The chemistry and physiology of ABA and its analogs is described by Milborrow (2). The naturally occurring enantiomeric form of ABA is (S)-ABA.

Plants have evolved various signaling mechanisms enabling them to withstand the multiple environmental conditions to which they are exposed, including variable water and nutrient availability, temperature variations, and excess ultraviolet-B light. Most of the plant responses to these abiotic conditions are mediated by abscisic acid. The ABA-signaling pathway is initiated by its binding to the specific receptor complex RCARs/PYR1/PYLs, leading to inactivation of type 2C protein phosphatases and activation of kinases, which mediate ABA-dependent changes in metabolism, gene expression, and ion channel activity (3, 4).

Interestingly, ABA plays essentially the same role in lower Metazoa (sponges and hydroids), where it mediates the responses of these sessile marine organisms to variations in water temperature and light. In sponges, the oldest Metazoa, their evolution dating back to 600 million years, temperature-signaling occurs *via* ABA (5). In *Axinella polypoides*, an increase of the water temperature results in an increased ABA synthesis and in the ABA-mediated stimulation of water filtration and oxygen consumption (6). Hydroids follow sponges in the evolutionary tree, having sensory, nerve, muscle and epithelial cells, a digestive cavity, and a rudimentary nervous system, but share with sponges a sessile lifestyle. In *Eudendrium racemosum*, light stimulates endogenous ABA synthesis and ABA stimulates stem cell-mediated tissue regeneration (7).

### ABA Origin and Role in Mammals

In the human body, ABA naturally originates from dietary sources and endogenous production through the carotenoid biogenesis pathway. ABA was first described in the brain of pigs and rats (8). The fact that animals fed a synthetic, ABA-free diet had ABA levels even higher than those of controls fed a vegetable diet was taken as an indirect proof that ABA was endogenously produced. To this point, a more recent observation allows the conclusion that ABA is indeed an endogenously produced signaling molecule: plasma ABA (ABAp) levels increase significantly in healthy humans after an (ABA-free) glucose load (9). On the one hand, this observation points to ABA as being endogenously synthesized; plus it suggests a role for ABA in the physiological response to glucose intake, a role until then shared by insulin and by the incretin glucagon-like peptide-1 (GLP-1).

Several *in vitro* observations support the conclusion that ABA is indeed endogenously produced by human and murine cells: granulocytes (10), monocytes and macrophages (11, 12), insulin-releasing cells (13), mesenchymal stem cells (MSCs) (14), hemopoietic progenitors (HP) (15), adipocytes (9), keratinocytes (16), and fibroblasts (17), all have been shown to produce and release ABA when exposed to cell-specific stimuli. The cell-specific functional effects activated by ABA in these cell types are in line with a conserved role of ABA as a signal relating cell response to changing environmental conditions. The fact that several different cell types can produce ABA implies that ABAp may derive from multiple sources. Markedly reduced ABAp levels were measured in patients with type 1 diabetes as compared with healthy individuals of similar age and body mass index (9), suggesting that β-pancreatic cells may be a relevant source of ABA. High glucose concentrations can stimulate ABA release from human adipose tissue biopsies *in vitro* (9) and the fact that adipose tissue accounts for a substantial amount of body weight, even in non-obese subjects, may make it another significant source of ABAp, in addition to pancreatic β-cells.

### Conservation of ABA-Signaling Pathway Hallmarks from Plants to Lower Metazoa and Mammals: G-Protein-Coupled Receptor, Protein Kinase A (PKA), cADPR, and Ca^2+^

One of the chief functions of ABA in plants is to reduce guard cell turgor, thereby contributing to the conservation of water during periods of drought. This functional response triggered by ABA is mediated by an increase of the cytoplasmic Ca^2+^ concentration $\left({\text{Ca}}_{\text{cyt}}^{2+}\right)$ in guard cells, induced by the intracellular Ca^2+^-mobilizing second messenger cyclic ADP-ribose (cADPR) (18, 19). cADPR is produced from NAD^+^ by ADP-ribosyl cyclases (ADPRCs), enzymes with an ancient evolutionary origin, being ubiquitously expressed from plants to lower and higher Metazoa (20).

Intriguingly, the signaling pathway downstream of ABA stimulating water filtration in sponges and tissue regeneration in hydroids shares with the plant ABA-mediated stomatal closure the same ADPRC-generated second messenger, cADPR (6, 7). ABA has been reported to activate the cyclase activity of *Arabidopsis* ADPRC, in the absence of protein synthesis, although the mechanism of activation was not elucidated (21). In *Eudendrium* and in *Axinella*, activation by ABA of the ADPRC occurs *via* a PKA-dependent phosphorylation. Indeed, the signaling pathway downstream of ABA in *Axinella* and in *Eudendrium* is similar and involves the sequential activation of PKA, phosphorylation and activation of ADPRC, cADPR overproduction, and increase of ${\text{Ca}}_{\text{cyt}}^{2+}$ (5, 7).

**Figure 1:**
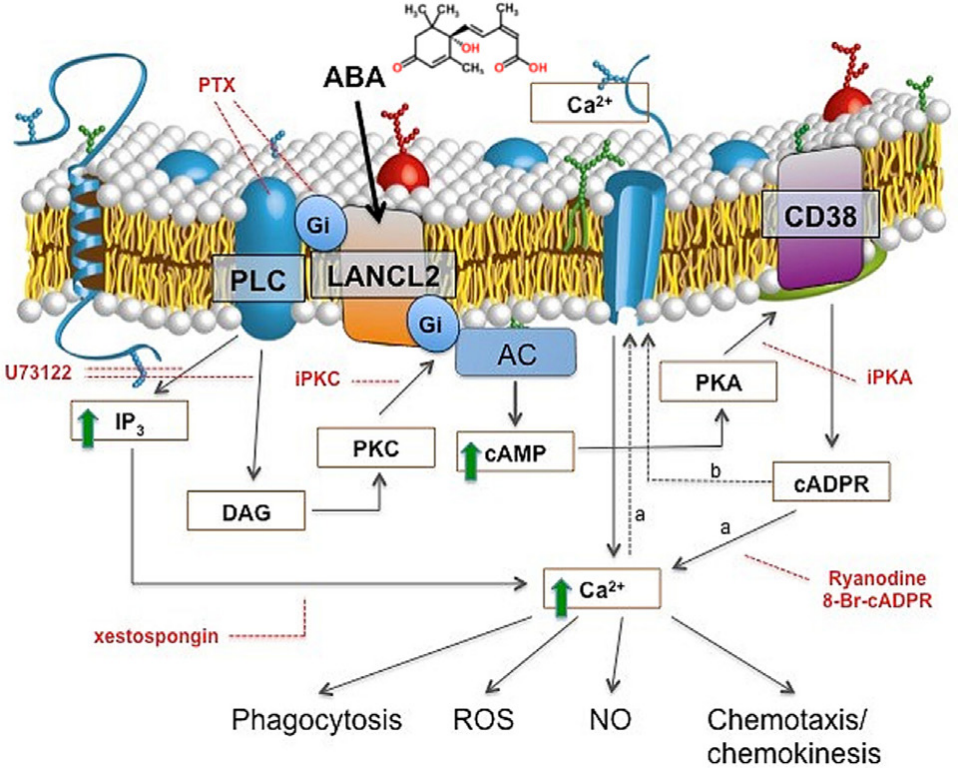
ABA signaling in human granulocytes. The interaction of ABA with a G-protein-coupled plasmamembrane receptor triggers: (i) activation of phospholipase C (PLC), overproduction of inositol triphosphate (IP_3_), and stimulation of a PKC-dependent adenylate cyclase (AC); (ii) activation of AC, overproduction of cAMP, protein kinase A (PKA)-mediated stimulation of ADP-ribosyl cyclase, and increase of [cADPR]_*i*_. Downstream of cyclic ADP-ribose (cADPR), two mechanisms (dotted lines) might cooperate to induce the observed increase of the [Ca^2+^]_*i*_: extracellular Ca^2+^ influx through store-operated Ca^2+^ entry (a), or, direct gating of a plasmamembrane Ca^2+^ channel by cADPR (b). Site-specific inhibitors of the ABA-signaling pathway are indicated in red. PTX, pertussis toxin; U73122, PLC inhibitor; xestospongin, IP_3_-specific Ca^2+^-channel blocker; I-PKA and I-PKC, PKA- and PKC-specific myristoylated (peptide inhibitors); 8-Br-cADPR, specific cADPR antagonist; Ry, Ryanodine (cADPR-specific Ca^2+^-channel blocker). The increased [Ca^2+^]*_i_* levels stimulate functional responses: phagocytosis, release of ROS and NO, chemokinesis, and chemotaxis to ABA.

The signaling pathway downstream of ABA in human granulocytes is strikingly similar to the one unveiled in lower Metazoa, and it involves an ADPRC (CD38) and its product cADPR (Figure 1). In addition to activating adenylate cyclase (AC), ABA also activates phospholipase C (PLC), with the consequent overproduction of inositol triphosphate (IP_3_); thus, the increase of ${\text{Ca}}_{\text{cyt}}^{2+}$ triggered by ABA in granulocytes is mediated by both cADPR and IP_3_ (Figure 1). The G-protein coupled to the ABA receptor was identified as Gi by its sensitivity to pertussis toxin (PTX) (10). The mechanism through which Gi mediates the activation of both PLC and AC was elucidated by means of transfection experiments, performed on human granulocytes with a chimeric G-protein, resulting from the fusion of Gαi with the last five aminoacids of Gαq (Gαq/i), and with transducin (αt), a scavenger of βγ subunits. Overexpression of Gαq/i increases the contribution of IP_3_ to the ${\text{Ca}}_{\text{cyt}}^{2+}$ rise induced by ABA and results in a fast and transient ${\text{Ca}}_{\text{cyt}}^{2+}$ increase, that becomes evident when the contribution of cADPR to the ${\text{Ca}}_{\text{cyt}}^{2+}$ rise is prevented by the cADPR antagonist 8Br-cADPR (22). Overexpression of αt dampens the ABA-induced ${\text{Ca}}_{\text{cyt}}^{2+}$ increase, demonstrating that the βγ subunits of Gi are responsible for the activation of both PLC and AC (22). A role for both cADPR and IP_3_ in the ABA-triggered increase of ${\text{Ca}}_{\text{cyt}}^{2+}$ has been reported in granulocytes (10) and in monocytes (12).

Conservation of the ABA-signaling pathway sequentially involving AC, cAMP, PKA-dependent ADPRC activation, cADPR overproduction, and ${\text{Ca}}_{\text{cyt}}^{2+}$ increase from sponges to lower and higher Metazoa points to its presence at a very early stage of evolution, in a precursor common to plants and animals. This may suggest an evolutionarily conserved role of ABA in adaptation to environmental stresses that has yet to be fully elucidated in humans.

### The Mammalian ABA Receptor: Lanthionine Synthetase C-Like 2 (LANCL2)

The *Arabidopsis* membrane-bound ABA receptor GCR2 shares a high amino acid identity with the mammalian peptide-modifying lanthionine synthetase C-like (LANCL) protein family (23), and the LANCL protein family in turn shows structural similarities with the prokaryotic lanthionine synthetase component C proteins (24) involved in the synthesis of lanthionine-containing antimicrobial peptides known as lantibiotics (25) such as nisin, which are used to prevent bacterial growth in foods. However, lantibiotics are not produced in animals; thus, mammalian LANCL proteins must have a different function than prokaryotic LanC proteins. The human genome contains three LANCL genes, *LANCL1, LANCL2*, and *LANCL3*, on chromosomes 2, 7, and the X chromosome, respectively (26, 27). In human epithelial cells overexpressing LANCL1 or LANCL2 fused to the green fluorescent protein (LANCL1-GFP and LANCL2-GFP), LANCL1-GFP is mainly found in the cytosol and in the nucleus, whereas LANCL2-GFP is associated with the plasmamembrane through N-terminal myristoylation (28). Recently, it has been demonstrated that the demyristoylation of LANCL2 by chemical or genetic means triggers its nuclear translocation. The nuclear enrichment of native LANCL2 was also induced by ABA treatment, suggesting that human LANCL2 is a non-transmembrane G-protein-coupled receptor susceptible to hormone-induced nuclear translocation (29).

Several lines of evidence support the conclusion that LANCL2 is a mammalian receptor for ABA. Sturla et al. (22) showed that LANCL2 was necessary for ABA binding and signaling in four different cell types (granulocytes, HeLa cells, and RIN-m and INS-1 cells) from two mammalian species: (i) silencing of LANCL2 abrogated the ABA-induced increase of the ${\text{Ca}}_{\text{cyt}}^{2+}$ and of cAMP in human granulocytes and also prevented the ABA-triggered functional response in these cells; (ii) LANCL2 overexpression conversely potentiated the ${\text{Ca}}_{\text{cyt}}^{2+}$ increase induced by ABA in granulocytes and conferred ABA responsiveness in terms of ${\text{Ca}}_{\text{cyt}}^{2+}$ and of cAMP increase to CD38^+^ HeLa; and finally, LANCL2 silencing abrogated the ABA-induced biochemical (increase of the [Ca^2+^]*_i_* and of the [cAMP]*_i_*) and functional (insulin release) responses in two different rat insulinoma cell lines. *In silico* and *in vitro* studies on the recombinant protein confirmed direct ABA binding to human LANCL2 (see ABA Improves Glucose Tolerance in db/db Mice Fed a High-Fat Diet).

### ABA Regulates Glucose Homeostasis in Mammals

Two groups independently hypothesized and experimentally validated the role of ABA in regulating glucose metabolism. Zocchi and colleagues followed an evolutionary reasoning: in plants, ABA lies at the crossroad between physical, nutrient, and parasitic stress responses. Its conservation in lower Metazoa suggested that it might have similar functions in higher Metazoa: indeed, ABA production occurs in human granulocytes (our first line of defense against pathogens) stimulated with physical or chemical stimuli and that ABA activates their defensive functions (10). That acute physical stress induces hyperglycemia and that the second messengers involved in ABA signaling in granulocytes, cAMP and cADPR, played a role in the signaling leading to glucose-induced insulin secretion (30, 31) suggested to explore the possible role of ABA in the arguably most important aspect of mammalian nutrient disposal, i.e., insulin release (13).

Bassaganya-Riera and colleagues were drawn to the study of the effect of ABA on mammalian glucose homeostasis based upon the structural similarities between ABA and the thiazolidinedione (TZD) class of insulin-sensitizing antidiabetic drugs, which bind to the ligand-binding domain of the transcription factor PPARγ and activate the transcription of several genes involved in glucose and lipid metabolism. They provided the first evidence *in vivo* that ABA exerts glucose-normalizing effects when administered to genetically obese db/db mice fed a high-fat diet (32) and to mice with diet-induced obesity (33). Moreover, ABA was shown to have similar efficacy to that of TZDs in mouse models of diabetes (32). However, ABA was shown to bind LANCL2 and to act independently of PPARγ (34). The next section will describe in more detail the *in vitro* and *in vivo* evidence accumulated until now, which confirms and expands our view of ABA as an endogenous regulator of glucose disposal in mammals and it highlights the fact that ABA is naturally present in our diet thereby providing a natural way to balance glucose levels in healthy individuals and manage glycemic control in prediabetic, diabetic, and metabolic syndrome patients.

## ABA: A Dietary Compound Present in Fruits and Vegetables

Many countries, including the United States, have a deficient intake of fruits and vegetables. On a country-wide scale, this results in a broad 1.5- to 2-fold reduction in consumption of phytochemicals (35). When individuals who meet fruit and vegetable recommendations are compared with those who do not, notable reductions in carotenoids (twofold), flavonones (threefold), and ellagic acid (fivefold) occur (36). These magnitudes of deficiencies have been linked to increased risk for the development of cardiovascular disease, metabolic disease, and cancer (37, 38).

Specifically, ABA is present in a variety of fruits and vegetables as shown in Table 1. The concentration varies depending on the type of fruit or vegetable but on average the concentration of ABA is 0.29/mg/kg wet weight of vegetable and 0.62 mg/kg of wet weight of fruit. According to the latest published dietary survey data from 2009 to 2012 (39), US adults aged 20 years or older consumed on average 1.05 servings/day of whole fruits and fruit juices (= 189 g/day) and 1.74 servings of vegetables per day including legumes and potatoes (229 g/day). Based on averages, a U.S. citizen of 20 years and older would consume 189 × 0.62 = 117.2 µg of ABA per day derived from fruits and 229 × 0.29 = 66.4 µg of ABA per day derived from vegetables. Thus, a US adult would consume an average of 184 µg of ABA per day derived from the 2.79 servings/day of fruits and vegetables.

**Table 1:** Concentration of ABA in various foodstuffs.

Concentration of ABA in various fruits and vegetables		
Food category	ABA level (mg/kg)	Reference
Fruits*—average total	0.62	
Apple	0.30	(40)
Apricot	0.32	(40)
Avocado	2.0	(41)
Banana	0.22	(40)
Bilberry	0.4	(40)
Citrus	1.25	(42)
Fig	0.72	(40)
Pepper fruit	0.25	(43)
Persimmon	0.10	(44)
Vegetables*—average total	0.29	
Barley	0.20	(45)
Cucumber	0.09	(46)
Maize	0.33	(47)
Pea	0.13	(48)
Potato	0.09	(49)
Soybean	0.79	(50)
Tomato	0.20	(46)
Wheat	0.15	(51)

*Fruits and vegetables are categorized according to Rehm et al. Vegetables including legumes, tomato, and potato.

The current recommendation is to eat ≥4.5 servings/day, which would lead to ≥297 μg of ABA per day in an ideal situation, but only 8% of the US adult population is meeting the dietary recommendations for fruits and vegetables of American Heart Association 2020 Strategic Goals (39). This means that 92% of the population is receiving an ABA-deficient diet according to these recommendations. It remains unclear what the ABA deficiency means in terms of long-term health effects, but it is tempting to speculate that the ABA deficiency might be a risk factor involved in the onset of diabetes and cardiovascular disease.

In a study designed to assess the effects of phytochemicals on health, an increase of combined fruit and vegetable intake by 480 g/day tripled the amount systemically available ABA after 6 weeks, identified ABA as one of three novel biomarkers (along with cyclohexadienecarboxylic acid and cyclohexanecarboxylic acid) and strongly correlated with decreased CVD risk biomarkers in a human trial (52). While a dietary adjustment of this magnitude may be unrealistic in the general population, a standard diet, with insufficient fruit and vegetable intake, clearly presents suboptimal ABA concentrations throughout the body. Currently, insufficient data on the necessity of phytochemical intake and the complexity of interactions among the 8,000 identified dietary bioactive compounds limit the ability to assign a recommended daily intake of ABA and similar compounds (53). However, by elucidating specific effects of its deficiency and benefits of its supplementation, ABA can become an accepted nutritional quantity like similar compounds, lutein and zeaxanthin with retinal health, for certain medical disorders associated with altered glucose metabolism and homeostasis.

## *In Vitro* Effects of ABA on Glucose and Lipid Metabolism

### ABA Stimulates Insulin Release from Pancreatic β-Cells and GLP-1 Release from Enteroendocrine Cells

High glucose concentrations stimulate production and release of ABA from rodent insulinoma cells and from human pancreatic β-cells (13). Nanomolar ABA in turn stimulates glucose-independent (i.e., in the absence of glucose) and potentiates glucose-dependent (i.e., in the presence of either low or high glucose) insulin release (13). The signaling pathway of ABA in β-cells involves its receptor LANCL2 (22), and the sequential activation of PTX-sensitive Gi, AC, cAMP production, PKA-mediated phosphorylation and activation of the ADPRC CD38, followed by accumulation of cADPR (13).

Oral glucose load stimulates insulin release from pancreatic β-cells principally *via* the incretin GLP-1, which is believed to account for up to 70% of glucose-stimulated insulin release (54). GLP-1 is produced and released into the blood by enteroendocrine cells (L cells) in response to high glucose concentrations in the gut. ABA induces glucose-independent GLP-1 release in the human L cell line hNCI-H716 through a cAMP/PKA-dependent mechanism and also enhances GLP-1 transcription (55). An increase of plasma GLP-1 (GLP-1p) is observed also *in vivo*, upon oral ABA administration in fasted rats (55). Interestingly, GLP-1 stimulates ABA release from an insulinoma cell line and from human islets, approximately 10- and 2-fold in low and high glucose, respectively (9). Altogether, these results suggest the existence of a positive feedback mechanism between ABA and GLP-1 activated by hyperglycemia, whereby ABA stimulates GLP-1 production by L cells and GLP-1 stimulates ABA release from β-cells.

The increases of the GLP-1 and ABA plasma concentrations that normally follow oral glucose intake are impaired in type 2 diabetes (T2D) (56, 57), further supporting the hypothesis of a mutual regulation between these molecules. Besides stimulating insulin release, GLP-1 has several beneficial effects on the cardiovascular system, including stimulation of cardiomyocyte glucose uptake *in vitro* and prevention of ischemic-reperfusion injury and improvement of myocardial performance *in vivo* [reviewed by Sarraju et al. (58)]. Thus, understanding the molecular cross talk between ABA and GLP-1 may shed new light on the physiology as well as the dysfunction of GLP-1-mediated cardiometabolic protection. In this respect, ABA administration improved atherosclerosis-induced hypertension, inflammatory immune cell recruitment into the aortic root wall, increased plasma triglyceride (TG) and non-esterified fatty acid concentrations, and upregulated aortic eNOS expression in ApoE^−/−^ mice (59), suggesting that ABA elicits a local antiatherogenic effect in the arterial wall of mice and supports its cardiometabolic protective role.

### ABA Stimulates Glucose Uptake in Rodent Adipocytes and Myoblasts

ABA at nanomolar concentrations stimulates glucose uptake by differentiated murine adipocytes and by rat myoblasts *in vitro*, to a similar extent as insulin at the same concentration (9). ABA increases glucose transporter 4 (GLUT4) translocation to the plasmamembrane in both cell types (9) and stimulates the Ser phosphorylation of Akt similarly to insulin (9).

Insulin-independent stimulation of GLUT4 translocation by ABA in adipocytes is particularly relevant to the glycemia-lowering effect of ABA. Mice with the adipose tissue-specific ablation of GLUT4, but normal GLUT4 expression in muscle, are insulin resistant and have an increased risk of developing overt diabetes (60); adipose tissue-specific GLUT4 KO mice are insulin resistant also in liver and muscle (61). Overexpression of GLUT4 in the adipose tissue can prevent diabetes in mice defective for muscle GLUT4 expression (61). Of note, LANCL2 facilitates phosphorylation of Akt by mTORC2 *via* direct physical interactions with both the kinase and the substrate (62). The active mTORC2 causes translocation of GLUT4 to the plasma membrane and glucose uptake in liver cells (63). Bassaganya-Riera et al. published for the first time that ABA treatment prevents LPS-induced downregulation of Glut4 in spleens of mice (34).

## ABA Ameliorates Glucose Tolerance and Adipose Tissue Inflammation

### ABA Improves Glucose Tolerance in db/db Mice Fed a High-Fat Diet

Bassaganya-Riera and colleagues demonstrated for the first time that ABA improves glucose tolerance in genetically obese db/db mice fed a high-fat diet and to mice with diet-induced obesity (32, 33). In another study, we demonstrated that ABA performs within 98% similarity to the market drug, Avandia, a PPARγ agonist. However, as opposed to TZDs, ABA does not bind to PPARγ (34). Of note, treatment with ABA helped reduce inflammatory markers in the adipose tissue. Specifically, treatment with ABA downregulated monocyte chemoattractant protein-1 (MCP-1) expression and the infiltration of pro-inflammatory F4/80^+^CD11b^+^ macrophages in the stromal vascular fraction of adipose tissue (34). Insulin has been shown to be less effective in improving glucose tolerance in an inflamed environment due to insulin resistance caused by inflammatory cytokines (64); however, the role of adipose tissue inflammation as a driver of insulin resistance is still debated. Furthermore, dietary ABA supplementation significantly reduced fasting insulin levels (65). While the benefits of ABA on glucose tolerance are clear, additional research into the impact and mechanisms of ABA in insulin resistance will enhance the functional knowledge on the actions of ABA in glucose metabolism.

### Microgram Amounts of ABA Improves Glucose Tolerance in Rats and in Humans without Increasing Insulinemia

Despite the strong evidence demonstrating that ABA can stimulate insulin release from pancreatic β-cells *in vitro* (13), subsequent *in vivo* studies revealed that low-dose ABA improves glucose tolerance without increasing insulinemia. Rats and healthy humans undergoing an oral glucose tolerance test (OGTT) and treated with ABA, at a dose comprised between 0.5 and 1 μg/kg body weight, both show an improved glycemic profile and lower insulin levels compared with untreated controls (40). This unexpected result may have several, not necessarily mutually exclusive, reasons: (i) stimulation of glucose transport in GLUT4-expressing cells by ABA may precede in time and/or exceed in extent the stimulation of insulin release; (ii) GLUT4-expressing cells may be more sensitive to the effect of ABA than β-pancreatic cells *in vivo*. In fact, the dose of ABA appears to be relevant for its effect on β-cells *in vivo*: at 50 mg/kg, oral ABA alone (without glucose) increases insulinemia and reduces glycemia in rats (55). There is growing consensus within the scientific community that the prolonged stimulation of insulin release from β-cells under conditions of chronic hyperglycemia contributes to their eventual demise (66). For this reason, antidiabetic drugs capable of lowering glycemia without increasing insulinemia are highly desirable.

### ABA Increases GLP-1p in Fasted Rats

Besides lowering glycemia and increasing insulin release (only when administered at the high concentration of 50 mg/kg), oral ABA determines the increase of GLP-1p, this effect having been tested only at high ABA concentration (55). The ABA-induced GLP-1p increase was measured both in the peripheral blood of rats pretreated with sitagliptin (an inhibitor of GLP-1 degrading enzymes), and in blood withdrawn from the portal vein of rats not pretreated with sitagliptin, clearly indicating that ABA stimulates also *in vivo* the release of GLP-1 from enteroendocrine cells, as it was demonstrated in an *in vitro* system. The increase in GLP-1p preceded the insulin increase (peaks at 20 and 40 min, respectively); thus, a positive feedback loop might exists between ABA increasing GLP-1 (55), which in turn determines a further release of ABA from β-pancreatic cells (9), which potentiate insulin release (13). The effect of low concentrations of ABA on the *in vivo* GLP-1 release remains to be determined. Given that a low ABA concentration failed to induce an increase in insulinemia, despite its glycemia-lowering effect (40), the physiological relevance of the depicted loop at low, endogenous, ABA concentration is negligible. It remains unknown whether other cell types are able to respond to ABA by releasing GLP-1, and whether ABA can stimulate GLP-1 secretion also acting as a hormonal stimulus on the vascular side of L-cells, as it happens for glucose (67).

Currently, T2D treatments aimed at inhibiting GLP-1-degrading enzymes (DPP4) or using GLP-1 mimetics are available (68). The fact that ABA stimulates GLP-1 release accounts for considering ABA as a new possible strategy to impact on the GLP-1 axis in T2D, alone or in combination with DPP4 inhibitor.

### ABA Reduces Adipose Tissue Inflammation

Obesity and diabetes are characterized by a low-grade systemic chronic inflammation and insulin resistance. Hypertrophic adipocytes are insulin resistant and secrete more free fatty acids and TGs than they take up, leading to lipid deposition in peripheral tissues such as the liver and skeletal muscle. These cells also produce chemokines, such as MCP-1, which promote macrophage infiltration into the white adipose tissue. Bassaganya-Riera’s team demonstrated that ABA treatment improves insulin sensitivity, decreases adipocyte hypertrophy, reduces macrophage infiltration into the white adipose tissue, and downregulates the levels of tumor necrosis alpha (TNFα) and MCP-1 in obese mice (32, 33). Later studies demonstrated that ABA, together with rosiglitazone, downregulates not only F4/80^+^CD11b^+^ pro-inflammatory macrophages in white adipose tissue (32), but also levels of MCP-1 (59). Interestingly, the downregulation of pro-inflammatory markers in ABA-treated mice could be explained by other findings, including the observation of upregulation of regulatory T (Treg) cells, together with a downregulation of blood CD11b^+^ CCR2^+^ (the ligand of MCP-1) monocytes (65). Furthermore, data demonstrated that with the increase in CD4^+^ T cells there was a corresponding decrease in the percentages of CD8^+^ T cells in MLN following dietary ABA supplementation. Additionally, MLN CD4^+^IL-10^+^ T cells and blood CD4^+^CD25^+^Foxp3^+^ Treg cells and CD8^+^IL-10^+^ T cells were significantly increased in ABA-fed mice. These results together support the hypothesis that ABA reduces obesity-related systemic inflammation, especially in the adipose compartment.

The abovementioned findings suggested that the beneficial effects of ABA on glucose tolerance were mediated, in part, by its anti-inflammatory actions. Mechanistically, the reduction in adipocyte hypertrophy can prevent the hypertrophic adipocyte-induced hypoxia within adipose tissue. HIF1α expression was identified to be downregulated by ABA treatment (34). Further, expression of HIF-inducible and -interacting genes, such as TLR4, JUN, and CDKN1A, were also identified to be downregulated by ABA treatment in splenocytes. Treatment with ABA also decreased TLR4 levels in blood and MLN (34). Examination of the HIF1α pathway in more detail may unveil additional mechanisms of ABA-mediated downregulation of adipose tissue inflammation.

### Impaired Ability to Increase ABAp after Glucose Load in T2D and Gestational Diabetes (GDM)

The increase of ABAp that occurs in healthy subjects after an oral glucose load (ABA response) (9) is impaired in patients with T2D and in women with GDM (56). GDM is a “reversible” diabetic condition that reverts to a normal glucose tolerant state after childbirth. Pointedly, normalization of glucose tolerance after childbirth was paralleled by restoration of both the ABAp response to oral glucose and normal fasting ABAp levels. Thus, impairment of the response of ABAp to hyperglycemia might be a commonality between T2D and GDM subjects, suggesting an important role for ABAp in maintaining normal glucose tolerance. Zocchi and colleagues also compared fasting ABAp before and after biliopancreatic diversion (BPD) in obese, but not diabetic subjects, and in obese T2D patients, in which BPD resulted in the resolution of diabetes. Compared to pre-BPD values, basal ABAp significantly increased 1 month after BPD in T2D as well as in NGT subjects, in parallel with a reduction of fasting plasma glucose, suggesting a beneficial effect of elevated ABAp on glycemic control (56). An intriguing hypothesis to explain the increase of ABAp after BPD is the stimulation of enteroendocrine cells by excess non-absorbed nutrients, resulting in an increased release of GLP-1 (69, 70), in turn stimulating ABA secretion from β-pancreatic cells.

The higher mean value and wider distribution of the fasting ABAp in the T2D subjects compared to the NGT controls (56) may reflect a pleiotropy of ABA-related dysfunctions in T2D, such as: (i) resistance to the glycemia-lowering effect of ABA, causing higher than normal basal ABAp levels, or, (ii) impairment of the molecular mechanisms regulating the increase of ABAp in response to hyperglycemia, causing ABAp levels to be in the normal range despite hyperglycemia. As opposed to what observed in T2D subjects, the fasting ABAp of the GDM subjects was consistently lower compared to the NGT controls, suggesting that insufficient ABA release in response to hyperglycemia may be the common mechanism of pathogenesis underlying ABA dysfunction in GDM. The fact that ABAp and its response to hyperglycemia are abnormal in T2D and GDM suggests a role for this dysregulation in the pathogenesis of these conditions.

## LANCL2 Signaling Pathway

### ABA Binding by LANCL2: *In Silico* and *In Vitro* Evidence

Direct evidence of specific and saturable ABA binding to recombinant, purified LANCL2 was obtained with two different LANCL2 recombinant proteins, a fusion protein with glutathione-S-transferase (LANCL2-GST) and LANCL2 cleaved from its GST tag, and through several different experimental approaches: binding with [^3^H]-ABA, scintillation proximity assays on immobilized LANCL2, dot blot experiments with biotinylated ABA (bio-ABA), and affinity chromatography of LANCL2 on immobilized ABA (71).

The relatively high Kd value for ABA of recombinant LANCL2 could be attributed to imperfect protein folding, as suggested by its fast precipitation after tag cleavage or to the requirement of posttranslational modifications to enhance its ABA-binding activity. Indeed, native LANCL2 is N-terminally myristoylated and interacts with membrane phosphatidylinositol phosphate(s), both modifications being required for its membrane association (28). Both ABA enantiomers, (S)- and (R)-ABA, completely displaced binding of (R,S)-[^3^H]ABA to LANCL2-GST, in agreement with the fact that both (S)- and (R)-ABA are biologically active on ABA-sensitive cells.

**Figure 2:**
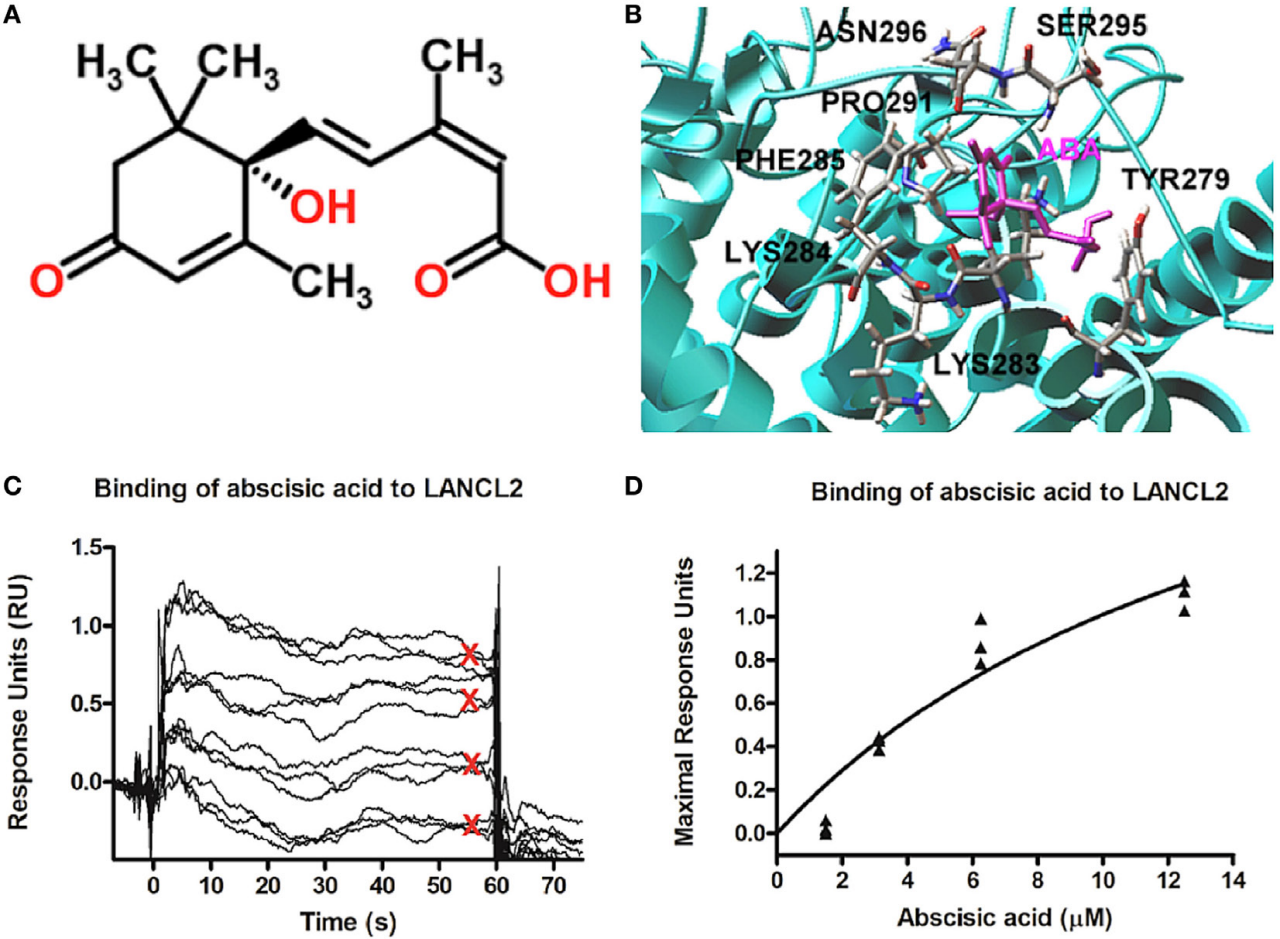
Model structure of lanthionine synthetase C-like 2 (LANCL2) and putative ABA-binding site. **(A)** Chemical structure of abscisic acid (76). **(B)** Representative binding modes of the most stable docked orientation of ABA (shown in pink) with LANCL2. The amino acid residues surrounding ABA are indicated. **(C)** Surface plasmon resonance sensograms for the binding of varying concentrations of ABA (1, 3, 6, and 12 µM) to immobilized LANCL2. **(D)** Plot of maximal resonance unit versus concentration of ABA. Steady-state dissociation constant was calculated to be 2.252 µM utilizing a 1:1 binding model. Binding data first shown by Lu et al. (72).

Parallel *in silico* studies, performed on a model structure of LANCL2, revealed a potential ABA-binding domain on LANCL2. This modeling prediction was validated experimentally using surface plasmon resonance (Figure 2) (72). Using that model, structure-based virtual screening was performed using compounds from NCI (National Cancer Institute) Diversity Set II, ChemBridge, ZINC natural products, and FDA-approved drugs databases. Lu et al. (73) identified several potential ligands using molecular docking. In order to validate the anti-inflammatory efficacy of the top ranked compound (NSC61610) in the NCI Diversity Set II, a series of *in vitro* and preclinical efficacy studies were performed in mice with inflammatory bowel disease (IBD). The results *in vivo* demonstrated that the lead compound, NSC61610, engaged LANCL2 in an AC/cAMP-dependent manner *in vitro* and ameliorated experimental IBD by downmodulating colonic inflammatory gene expression and favoring Treg cell responses (73). New derivatives and analogs of ABA and NSC61610 that bind to LANCL2 have been developed as potential, pharmaceutical activators of the LANCL2 pathway. BT-11 is being developed as a first-in-class oral small molecule therapeutic for Crohn’s disease (74, 75). BT-11 is an orally active, locally acting small molecule compound that binds to LANCL2 in the gastrointestinal (GI) tract and decreases leukocytic infiltration, mucosal thickening, and epithelial erosion in the GI tract. These histological improvements correlate with decreased numbers of inflammatory F4/80^+^CD11b^+^ macrophages and effector T helper 1 (Th1) cells in the gut mucosa. BT-11 also increases the levels of FOXP3-expressing CD4^+^ Treg that express IL-10 in the gut lamina propria, spleen, and MLN. Gene expression analyses confirmed that oral administration of BT-11 upregulates the expression of IL-10 and LANCL2, and downregulates the expression of TNFα GI tissue of mice with experimental IBD. Furthermore, PK studies with BT-11 showed low plasma concentrations and elimination within hours after oral administration. Plasma concentrations were only slightly higher after oral administration of 500 mg/kg than they were after 80 mg/kg (sixfold dose difference). BT-11 concentrations in the intestine after the 80 mg/kg treatment were considerably higher than concentrations measured in plasma at the same time. In addition, BT-11 gave consistent 90% IBD reduction in three mouse models after both oral and rectal administration (8 mg/kg).

### Membrane Localization of LANCL2 and Mechanism of ABA Transport through the Plasma Membrane

Lanthionine synthetase C-like 2 anchoring to the plasmamembrane occurs *via* N-terminal myristoylation and also *via* a basic phosphatidylinositol phosphate-binding site (28). LANCL2 is not a transmembrane protein, as it is detached from the erythrocyte membrane by relatively mild chemical treatments, in the absence of detergents (77). Thus, LANCL2 localization implies that ABA binding may occur intracellularly, raising the issue of how ABA gains access to its intracellular receptor. Protonated ABA can diffuse through the lipid bilayer and this passive diffusion is believed to drive ABA out of the xylem sap and into plant cell tissues, although transmembrane, ATP-dependent ABA transporters are also present in plant cells (78). The pKa of ABA is 4.7; thus at pH values typical of extra- and intracellular fluids in animals (approximately 7.5) <0.2% of ABA is protonated and ABA is mostly membrane impermeable. The protein responsible for ABA transport was identified as the anion transporter Band 3 in human erythrocytes (RBC), on the basis of several lines of evidence: (i) chloride ions compete with ABA for influx into RBC; (ii) the specific Band 3 inhibitor DIDS inhibits influx of ABA; (iii) Band 3 binds bio-ABA and bio-ABA can be displaced by excess ABA, indicating a specific interaction between ABA and the transporter; (iv) proteoliposomes reconstituted with purified Band 3 are able to transport ABA and sulfate (77). The erythrocyte anion transporter Band 3 (also called AE1) is a member of a protein family of anion transporters that includes AE2 and AE3. While AE1 is expressed only on erythrocytes, AE2 and AE3 are expressed in nucleated cells. In line with what was observed on Band 3, AE2 has been recently shown to mediate ABA transport in human nucleated cells and to allow the functional effect of extracellular ABA in keratinocytes (79).

ABA transport across AE1 and AE2 is bidirectional, allowing the free flux of ABA along its concentration gradient. The non-concentrative nature of the transporter allows circulating or paracrinally produced ABA to exert its functional effect on target cells: ABA-releasing cells can export ABA to bystander cells or into the bloodstream and ABAp can reach cells distant from the ABA-producing ones. Indeed, autocrine (9–11, 16, 17), paracrine (12, 15–17), and endocrine (13, 40, 55) effects of ABA have been described.

### ABA/LANCL2 Signaling in Cells Involved in Glucose Homeostasis: Adipocytes, Myocytes, and Hepatocytes

The first evidence indicating that ABA, similar to insulin, triggers the Ser phosphorylation of protein kinase Akt, came from experiments on adipocytes, as already described above (9). Later, by the use of LY294002, a PI3K specific inhibitor, the PI3K/Akt signaling pathway was demonstrated to mediate the ABA-induced activation of NADPH oxidase and of ROS generation, in the murine macrophage cell line RAW264.7 (11) and of glucose transport in rodent 3T3-L1 adipocytes (80). More recently, Zeng et al. (62) demonstrated that LANCL2 positively regulates Akt phosphorylation in four different liver cell lines. This regulation does not occur through the canonical-insulin signaling pathway, since LANCL2 knockdown affected neither the level of insulin receptor nor its phosphorylation. Instead, LANCL2 was found to physically interact with Akt. Through *in vitro* kinase assays with immunoprecipitated mTORC2 (one of the kinases having Akt as substrate) and recombinant Akt, in the absence or presence of purified LANCL2, Zeng et al. (62) demonstrated that LANCL2 regulates Akt phosphorylation by mTORC2. In addition, LANCL2 facilitates the phosphorylation of SGK1, and in turn of NDRG1, by mTORC2. Whether LANCL2 physically interacts with SGK1, as a common mechanism with Akt, in order to facilitate its phosphorylation by mTORC2, remains to be determined. Given the established role of ABA and of Akt in glycemia regulation, further investigation on the molecular interaction between LANCL2 and Akt in cells other than liver cells are needed.

### ABA/LANCL2 Signaling in Immune Modulation

ABA expands human uncommitted HP *in vitro*: 24-h priming with low micromolar ABA prior to seeding of the cells in a growth factor-enriched medium stimulates colony output from human cord blood-derived colony forming cells (CFC) and also from purified CD34^+^ cells, the subpopulation of HP containing the most immature progenitor cells. ABA stimulates the proliferative potential of the single CFC, increasing cell number in first generation colonies, and also stimulates the self-renewal of clonogenic HP, increasing the number of second and third generation colonies and resulting in a 1-log higher cell expansion factor in the ABA-treated cells compared with controls (15).

The stimulatory effect of ABA on HP proliferation is mediated by an increase of the ${\text{Ca}}_{\text{cyt}}^{2+}$, which is dependent upon a signaling pathway sequentially involving a PTX-sensitive Gi, stimulation of the ADPRC activity of CD38 and increase of intracellular cADPR (15). The ABA-induced increase of the ${\text{Ca}}_{\text{cyt}}^{2+}$ exerts transcriptional effects on HP *via* the nuclear translocation of NF-kB, stimulating transcription and release of several cytokines and growth factors, including VEGF, IL-8, IL-1β, IL-9, Fas ligand, GRO, and RANTES. VEGF and IL-8 are partly responsible for the autocrine stimulation of cell proliferation induced by ABA on HP, since blocking antibodies against these cytokines reduce the ABA-mediated increase of colony output. Interestingly, IL-8 stimulates release of ABA from MSCs (14).

**Figure 3:**
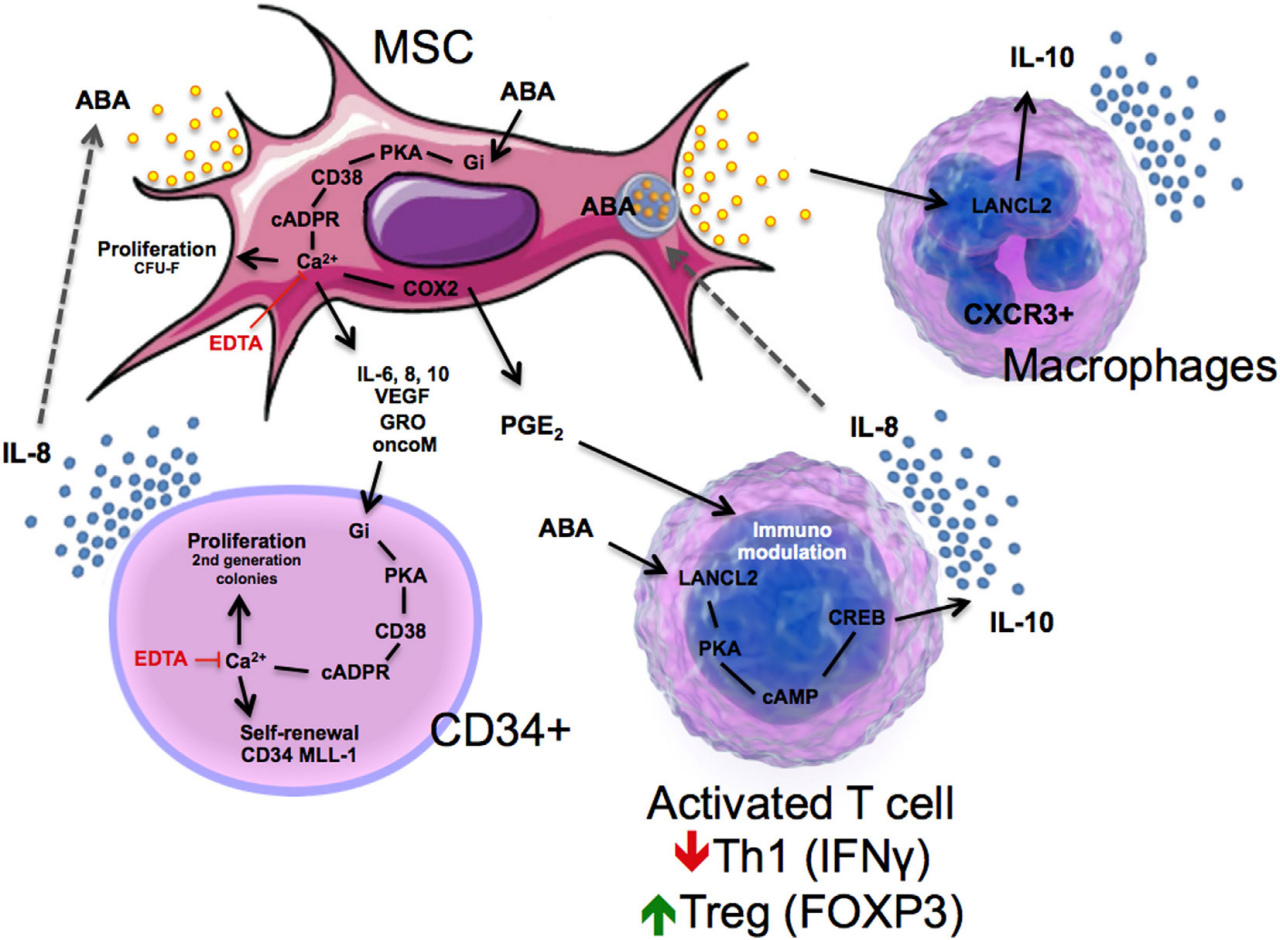
Schematic representation of immune modulatory actions of ABA in mesenchymal stem cell (MSC), CD34^+^ cells, T cells, and macrophages through lanthionine synthetase C-like 2 (LANCL2)-dependent mechanisms.

In the hemopoietic niche, MSCs provide physical and trophic support to HP, and function as immunomodulators, preventing lymphocyte activation, which negatively affects hemopoiesis. Indeed, MSC co-transplantation is a routine practice in allogeneic hematopoietic stem cell transplantation in some hematological centers (81). Several cytokines released by activated allogeneic lymphocytes, TNF-α, RANTES, and IL-8, induce the release of ABA from human MSCs; ABA in turn stimulates MSC proliferation and the release of hemopoietic growth factors, including IL-6, IL-8, oncostatin M, MIP-1d, and GRO (14). Presence of an anti-ABA monoclonal antibody prevents the growth-stimulatory effect of the coculture with MSC on second generation colony output from CD34^+^ cells (15). Thus, a positive feedback mechanism can be envisioned between MSC and HP in the hemopoietic niche, where ABA released by MSC behaves as an autocrine stimulator of stromal function and as a paracrine growth factor for HP (Figure 3).

ABA is also released by activated granulocytes (see Figure 1), eliciting the intriguing hypothesis that ABA may function as either a “danger” signal during infection or inflammation or a regulatory molecule for maintaining immune homeostasis. Such series of events are intimately linked to activation of LANCL2, which triggers a decrease in F4/80^+^CD11b^+^ macrophages, plus an increase in circulating Treg cells after dietary ABA treatment (65). The activation of LANCL2 also triggers a release of IL-10 in CXCR3^+^ macrophages (Figure 3). Linked to these findings, Bassaganya-Riera et al. (34) published a study demonstrating that dietary ABA supplementation reduced levels of TNFα while upregulating Glut4 in the spleen during LPS challenge of mice. These results have been validated by another study in which activation of LANCL2 led to a decrease in TNFα, MCP-1, and IFNγ, as well as a reduction in Th1 CD4^+^ T cells and an increase in Treg cells by the novel LANCL2 ligand, BT-11 (75). BT-11 binds to LANCL2, is orally active, has demonstrated efficacy in three validated mouse models of IBD at 8 mg/kg, and has a benign safety profile based on single dose, multiple dose and DRF studies in rats up to 1,000 mg/kg (74). Thus, activation of LANCL2 acts as an internal sensor activated by environmental dysregulation (e.g., inflammatory, dietary, or metabolic signals) that activates cellular and molecular pathways involved in homeostatic control of immunity and metabolism.

## Conclusion

ABA and its proposed receptor LANCL2 are emerging as new important players in the physiology and in the dysfunction of mammalian glucose homeostasis and immunoregulation. Over 28.3 million Americans have T2D and over 40.1% of middle-aged adults have prediabetes, a disease that has reached pandemic proportions in the U.S. Diabetes is the seventh leading cause of death worldwide, mainly due to its cardiovascular sequel. While T2D is an irreversible condition, prediabetes is not. Only less than 4% of prediabetic subjects receive pharmacologic treatment, as they do not yet classify as “diabetic.” Prediabetic or high-risk individuals, such as those afflicted by excess body weight, high blood lipids and hypertension, all hallmarks of the metabolic syndrome, are advised to follow dietary and lifestyle guidelines, which have a very low rate of adherence. Consequently, many of these subjects will become diabetic. This dramatic forecast calls for new and aggressive strategies to tackle the expanding incidence of diabetes pandemic before it becomes an even greater societal burden.

*Dietary low-dose ABA* improves glucose tolerance in healthy subjects without increasing insulinemia and oral ABA improves glucose tolerance in diabetic mice. Furthermore, the increase of ABAp that occurs after an OGTT is impaired in patients with T2D and in women with GDM. The mechanism of action of low-dose ABA *in vivo* is likely mediated by its stimulation of GLUT4-mediated glucose uptake, rather than insulin release, as insulinemia is in fact reduced by ABA treatment. Dietary ABA administration in the form of foods or beverages can be proposed as a new intervention, capable of improving glucose tolerance, while at the same time sparing the β-cell reservoir. ABA is both an endogenous and dietary LANCL2-activating compound. Nanomolar plasma concentrations, in the physiological range and attainable by dietary intake of a dose of approximately 1 µg/kg body weight, are effective on glycemic control. These two facts together allow its safe use to maintain healthy blood glucose levels and to prevent diabetes. In addition, low-dose ABA does not increase insulin release and this may prove advantageous in diabetic, prediabetic, and metabolic syndrome subjects, who are deficient in and/or resistant to insulin. By reducing the chronic stimulation by hyperglycemia of β-cells to release insulin, low-dose ABA administration should improve survival and function of these cells.

Identifying other *LANCL2-activating compounds* may also prove a successful strategy, in view of the multiple anti-diabetic, anti-inflammatory, and immunoregulatory effects that they might trigger. Indeed, LANCL2 can: (i) facilitate Akt phosphorylation and increase GLUT4-mediated glucose uptake, an Akt-dependent mechanism; (ii) mediate the ABA-stimulated GLP-1 release from L cells; and (ii) exert immunoregulatory and anti-inflammatory actions in the gut, blood vessels, and adipose tissue. The potential role of LANCL2 as a new drug target in diabetes has already been suggested (82) and efforts at discovering LANCL2-targeting drugs are underway. Furthermore, the ABA-based technology through the LANCL2 pathway to fight inflammation and diabetes has been patented, through method of utilization as well as composition of matter claims, by Bassaganya-Riera et al.

In summary, the data reviewed in this manuscript demonstrates that dietary or endogenously produced ABA is a new human signaling molecule that regulates responses to environmental challenges (i.e., metabolic, nutritional, inflammatory, and immunological), and it is implicated in glycemic control through a LANCL2-dependent mechanism. ABA not only regulates blood glucose levels and prevents the onset of T2D in mice and rats, but more importantly microgram amounts of dietary ABA are sufficient to improve glycemic controls in humans. Given the extensive results demonstrating a role for ABA in regulating glycemic control, ABA-based interventions have the potential to improve glycemic control in millions of people afflicted by prediabetes, diabetes, and metabolic syndrome worldwide.

## Methods

This comprehensive and up-to-date review summarizes the *in vitro*, preclinical, mechanistic, and clinical results obtained over the past 15 years by the two leading groups involved in the study of the metabolic, nutritional, and immunological actions of ABA. Only two such reviews exist on this subject (83, 84); with the new review, the authors wish to implement the literature with their most recent results, focusing on the arguably most clinically relevant role of ABA as a new human hormone involved in the control of glycemia. The authors kept a balance between independently published papers and papers published by the authors. The scientific literature summarized in the review has been published over the years 2001–2017 in peer-reviewed international journals, including Proc Natl Acad Sci U S A, J Biol Chem, FASEB J, Stem Cells, Clin Nutr, J Nutr Biochem, J Cell Physiol, and PloS One, among others.

## Author Contributions

EZ, JR, AL, and RH designed the architecture of the review. EZ, SB, NP, RH, JR, AC, NT-J, VZ-R, LS, and AE performed the searches for the review. EZ, SB, RH, AC, JR, and AE wrote and edited the manuscript.

## Conflict of Interest Statement

RH and JR hold patents for the use of abscisic acid in treating disease. The other authors declare no conflict of interest.

## Abbreviations

ABA, abscisic acid; cADPR, cyclic ADP-ribose; cAMP, cyclic adenosine monophosphate; GLP-1, glucagon-like peptide-1; GLUT4, glucose transporter 4; LANCL2, lanthionine synthetase C-like 2; PKA, protein kinase A; TZD, thiazolidinedione.
